# “What do You Mean I’ve Got to Wait for Six Weeks?!” Understanding the Sexual Behaviour of Men and Their Female Partners after Voluntary Medical Male Circumcision in the Western Cape

**DOI:** 10.1371/journal.pone.0133156

**Published:** 2015-07-15

**Authors:** Yoesrie Toefy, Donald Skinner, Sarah C. Thomsen

**Affiliations:** 1 Karolinska Institutet, Department of Public Health Sciences, Stockholm, Sweden; 2 Stellenbosch University, Department of Interdisciplinary Health Sciences, Tygerberg, Cape Town, South Africa; London School of Hygiene and Tropical Medicine, UNITED KINGDOM

## Abstract

**Background:**

Several studies have shown that voluntary male medical circumcision (VMMC) reduces the incidence of the Type-1 human immunodeficiency virus (HIV) in heterosexual men by up to 60%. However, there is an increased risk of transmission of STIs, including HIV, in the immediate post-operative period after receiving VMMC. This study is to understand sexual practices of couples in the post-operative period in a Coloured population in the Western Cape Province of South Africa.

**Methods:**

Coloured Males who had undergone VMMC in the previous six months in the Cape Town area and their partners participated in eight single-gender focus group discussions. The groups explored why the men decided to undergo VMMC, what kind of counselling they received, and how they experienced the 6-week post-operative period, including sexually.

**Results:**

The primary motivation to VMMC uptake included religious injunction and hygiene reasons and protection against sexually transmitted infections not necessarily HIV. There was some exploration of alternative sexual practices. During the period immediately post operation the respondents spoke of pain and fear of any sexual arousal, but towards the end of the six week period, sexual desire returned. Both men and women felt that sex was important to maintain the relationship. Gaps were identified in the pre- and post-MC procedure counselling.

**Conclusions:**

There is a real risk that men in this population may begin sex before complete healing has occurred. VMMC counselling to encourage men to stay sexually safe in the wound-healing period, needs to take into account the real-life factors of the circumcised men. It is essential from a public health, and gender perspective that effective counselling strategies for the VMMC post-operative period, and the longer term, are developed and tested.

## Background

### Background and context

South Africa holds the dubious title of being the country with the highest number of HIV positive individuals–over five million. [1] The Coloured community, which accounts for 48.8% of the Western Cape’s population [2] has a growing HIV prevalence rate—7.6% according to the latest ante natal data. [3] The heightened HIV risk to this population group lies in a high illicit drug and alcohol use in the community, which is associated with risky sexual behaviour [4–6].

Due to its known protective effects [7–9], the South African Department of Health has made a commitment to rolling out voluntary medical male circumcision (VMMC) in all provinces [10–11]. Thus far, about 3,600 medical circumcisions have been performed at the government’s Male Circumcision clinics since the start of the provincial VMMC rollout at the end of 2010 until April 2013 in the province. The Xhosa community, who make up 21% of the population in the province, practice traditional circumcision as part of the initiation of boys into adulthood and therefore consider medical circumcision outside their cultural norms [12]. There is little knowledge about the perceptions of VMMC among the Coloured population in the Western Cape. What is known is that the Coloured community do not consider HIV as a disease that affects their community [6, 13–14]

Sexual contact carries an increased risk of transmission of STIs, including HIV, in the immediate post-operative period after receiving VMMC. However, it appears that resumption of sex after the VMMC procedure is not unusual. In an observational study in Nyanza, Kenya, 30.7% of all participants and 65.7% of married or co-habiting participants, resumed sexual intercourse before wound healing, usually in the first 3–4 weeks after VMMC, despite counselling [15]. In the study conducted in Kisumu, Kenya, they also found the risk factors for sex before healing were being married or having 2 or more sex partners in the last year [16]. Similarly, in Zambia, 24% of men reported resuming sex early, 46% of which did so in the first three weeks [17]. In the Rakai, Uganda VMMC trial, about 11% of HIV+ and HIV- participants reported having intercourse before certified wound healing, despite intensive pre- and post VMMC counselling and despite the men knowing their HIV status via the HIV test they received as part of their Standard of Care package. Female partners of HIV infected trial participants were 3.5 times *more* likely to acquire HIV if the couple resumed sexual intercourse early than the partners of those who did not receive VMMC (the control group), causing the authors to conclude that “…strict adherence to sexual abstinence during wound healing and consistent condom use thereafter must be strongly promoted when HIV-infected men receive circumcision.” Ironically, the risks of sero-conversion to HIV due to early resumption of sex after MMC seem to be especially high for women in the short term [18]. In fact, Hewett et al found that the model estimates that of the 61 000 men circumcised in one year, early resumption of sex leads to 69 extra HIV infections (32 among men, 37 among women), but estimates a net effect of 230 fewer HIV infections in one year, predominantly among men. 16], Recommendations have been made for developing and evaluating optimal counselling strategies among men seeking VMMC and to assess the effectiveness of behaviour change communication strategies [19]. One intervention, consisting of a 3-hour behavioural change component, has shown to be effective in preventing risk compensation behaviour three months after VMMC [20]. However, to our knowledge there is little scientific data about what works in the immediate post-operative period. In addition, there is a recognition that lack of human resources presents a barrier to the provision of such intense services, particularly if repeated messaging is to occur. Clearly, more innovative strategies for communicating with, and effectively altering behaviour in, men and their partners in the post-operative period of VMMC are needed in South Africa. As part of this development more knowledge is needed about current users of VMMC in order to find the best messages to include in these innovative strategies.

The primary objective of this study was to seek some understanding on why men in a predominantly Coloured community of South Africa, sought VMMC and to document their experience of the post-operative period in terms of penile recovery. Additionally, we wanted to understand the reasons why men might resume sex early after the VMMC procedure. Finally, we also wished to explore strategies that couples employ to negotiate the 6-week recovery period. The aim was to influence the counselling strategies of the VMMC programmes in this area in order to promote healthier sexual behaviour after the VMMC procedure. The term ‘healthier sexual behaviour’ is meant to cover both abstinence and non-penetrative sex.

### Methodology

A qualitative approach using focus groups discussions was used to develop a better understanding of how men and their partners feel about VMMC and sexual patterns around this time period.

#### Setting

The research was done in catchment areas of the Heidevelt Public Health Clinic and Mitchells Plain Hospital in Cape Town, in the Western Cape Province of South Africa. The study sites were chosen in conjunction with the provincial health department. The communities in the catchment area of the two selected township areas are almost exclusively Coloured, Afrikaans-speaking. The term Coloured refers to an official South African race group that is predominantly mixed ancestry that is used in research and census data. There is a large Muslim population (10–15%) in the catchment area for one of the townships. Both areas are densely populated with a low socio-economic base. The housing is typically one to two-bedroom” maisonette” housing with an average population density of 9,600 per km^2^. In 2011, the population of the suburb Mitchells Plain was 310 485 and the average household size was 4.57 [2].

The two healthcare institutions from which the sample was drawn have been offering VMMC as a form of protection from HIV for men since 2011. The clinics operate one morning a week on an appointment-only basis.

#### VMMC counselling procedures

On the day of the operation, the patient is asked to come in early for a mandatory HIV test including pre- and post-test counselling. If the patient tests positive, then the circumcision procedure is deferred until he can produce a CD4 count above the ARV admission point. The surgical procedure follows a strict protocol developed by the South African National Department of Health with the assistance of the Medical Male Circumcision task team including, South African National AIDS Council, UNAIDS, World Health Organization and others. [21] A very brief post-operative counselling session is conducted with the patient before he is discharged with information on how to care for the wound, on avoiding penetrative sex during the six week recovery period, and on the necessity for condom use for safe sex after wound healing.

#### Sample

The study included six focus groups with men and three with women (Table 1). The sampling was done in conjunction with the booking officers at the two clinics. Male participants were drawn from their theatre records of the last six months. The records consist of monthly lists of names, ages and contact details of VMMC recipients over the past six months. The fieldworker contacted all men on the clinic lists starting from the earliest month to the latest. Three men refused to participate at the initial contact point, claiming time-constraints, and five men who initially agreed to participate, did not arrive at the group sessions. No information is available on them and other than citing time constraints, no other reason for not coming were given.

**Table 1:** The study participants of the focus groups by sex and religion.

No.	Gender	Number of Participants	Age Range	Religion (Muslim; Christian)
1	Male	6	19–36	4;2
2	Male	6	22–45	3;3
3	Male	6	23–63	4;2
4	Male	7	18–42	5;2
5	Male	6	20–39	4;2
6	Male	7	21–53	4:3
7	Female	4	22–41	2;2
8	Female	5	19–34	3;2
9	Female	5	25–52	3;2

At the end of each focus group, the facilitator asked the participants the names and contact details of their sexual partners over the six-week post-operative period. Of the 38 men who participated, 26 men gave details of their partners. Reasons for not giving the details of their partners ranged from not knowing where their ex-partners are to the stated conviction that their partners would not participate in focus groups. No men reported having a male partner. The researcher contacted these partners and ended up with three focus groups with women who were partners of men who had recently undergone VMMC. Seven women refused to participate (time constraints, personal reasons) and five women did not arrive at the sessions. No additional information is available on those who did not participate.

All of the participants had been residents in the community for most of their lives and were Coloured. The population of the clinic’s catchment area is largely of the ‘Coloured’ race group and also 10–15% Muslim.

#### Interview schedules

The interviews covered issues around the reasons why they chose to be medically circumcised as adults, what the experience of the actual procedure entailed, and how they coped with any discomfort or pain during the initial recovery period. The interview then delved into the coping mechanisms employed later in the six-week period, particularly around the issues of desire and sex. The interview attempted to investigate the cultural importance of maintaining a sexual relationship with a regular partner.

The interviewer, who is first author, is skilled in doing qualitative interviews and was key in developing the research instruments. He is a resident of Cape Town who has done other research projects in similar communities. This was felt to be important as fairly sensitive topics were allowed to be explored fully by the participants.

All interviews were conducted in Afrikaans, tape recorded and then transcribed and translated into English.

#### Analysis

A contextualized content analysis approach was used to analyse the data [22]. All the interviews were read several times by the authors so that a familiarity with the material could be established. On the basis of this familiarity, a set of themes were drawn out revolving around reasons for seeking VMMC, the experience around the procedure, the impact (short and long-term) of the procedure on relationships and sexual behaviours. The first author used these themes to code the interviews with the assistance of Atlas.ti. The themes formed the basis for the analysis presented below. Once the provisional analysis had been done all the interviews were reread, as a validity measure, to check for contradictory findings, and if any information had been inadvertently excluded. In the analysis we acknowledge that using a male interviewer could have impacted the validity of the responses of the female participants. We have taken this into account in the analysis.

#### Ethics

The study was approved by the Stellenbosch University Health Research Ethics Committee (Reference Number: N13/02/018) and was accepted by the Western Cape Department of Health (Reference Number: RP 100/2013). The identity of all participants remains confidential. All respondents gave written informed consent prior to the interviews. All transcripts have had all personal details of the respondents removed. Copies of all the interviews are held only on password protected computers.

## Results

Although the subject matter was culturally sensitive and potentially emotive, the male participants were surprisingly candid and open to the questions asked. The female participants were much more inhibited, possibly because the facilitator was a male.

### Reasons for seeking VMMC

All the Muslim men who underwent VMMC did so as a precondition for converting to Islam and the majority did so to marry Muslim women. There is a strong religious and cultural directive to circumcise Muslim male infants in the first three weeks following birth. This directive extends to men entering Islam as adults. So for many the decision was due to external family pressure and not a decision taken to protect themselves or their partners. They may not even have been aware of the protection component before they requested the service. In these cases their partners may not have even been involved in the decision making as it is a prescribed duty to convert (and be circumcised) if the men were to marry a Muslim woman.

*“… so I agreed to convert to Islam and her father insisted that I get a “soennat” [A Malay culture word meaning circumcision]*.*”(Male*, *Muslim*, *23 years)*

*“… [but] I would have never considered doing it if I didn’t have to convert to Islam*.*” (Male*, *Muslim*, *20 years)*

All of the Christian participants cited cleanliness as a reason for seeking VMMC. The explanations centred on the presence of dirt or leftover material such as sweat or semen, the bad smell and the additional need for cleaning. This was found to be repulsive to both parties, but especially to the female partners (including Muslim females).

*“It’s true about the smell*. *I have always been aware of my smell and no matter how many times I washed and cleaned behind the foreskin that smell would always be in my nose*.*” (Male*, *Christian*, *27 years)*

*“You know*, *all that dirt that sits under the skin*. *It’s not nice…” (Female*, *Muslim*, *19 years)*

None of the participants gave HIV as a reason for seeking VMMC. Comments in the groups indicated that they felt safe from HIV, that it belonged to other areas of the country or to other racial communities. The Coloured community has historically had a lower level of HIV infection, and do not see themselves as vulnerable. There is a strong undercurrent of racism and stigma in the community as HIV is seen as a ‘Black’ disease or a disease that belongs to other distant communities. As indicated earlier, however, this prevalence level is changing [3].

*“In my case*, *not really*. *It’s just that those things are not really in mind*, *you know*. *It’s nice to know that it does…” (Male*, *Christian*, *51 years)*

*“It’s not important to us here in Cape Town*, *perhaps there in KwaZulu-Natal…” (Female*, *Christian*, *34 years)*

Some participants gave health reasons for undergoing the procedure. While not relating to HIV specifically, the connections were made to other sexually transmitted infections (STIs). This arose from both the male and female respondents.

*“Well*, *in my case*, *it was a factor because of my past history with STIs*.*” (Male*, *Christian*, *42 years)*

*“I’m actually the reason why my husband went for the operation…My bladder has always an infection in it*.*” (Female*, *Muslim*, *29 years)*

Besides religious influences and partners pushing them, pressure also came from their doctors or clinic nurses when they presented with STI-related symptoms.

*“…and I was actually forced by my doctor to do it because for as long as I can remember*, *I have been having problems down there*.*”(Male*, *Christian*, *47 years)*

### Issues arising in the post-operative period

Participants reported on the issues arising in the post-operative period as being different depending on how much time had elapsed. Therefore, we present the results here in different periods: 1) the first two weeks, 2) weeks 3–4, and 3) weeks 4–6.

A few participants spoke about the pain or discomfort during the operation and until the aesthetic wore off, although most seemed surprised at the simplicity of the procedure.

*“The actual op wasn’t that sore*. *It was quick” (Male*, *Muslim*, *31 years)*

Discussions about the first two weeks were primarily centred on recuperation and health-related issues. Respondents spoke particularly about how painful this period was. The respondents found this time period very difficult to cope with and struggled to deal with the discomfort and pain. It was treated with some humour during the discussion, but at the time they clearly struggled. There was no consideration of sex and arousal caused an escalation in pain.

*“I’m not going to lie*. *It was bloody sore*. *When the medicine wore off*, *it was paining like hell*.*” (Male*, *Muslim*, *18 years)*

*“(laughs) He was like a baby for more than two weeks*, *shame…” (Female*, *Christian*, *43 years)*

The next two weeks (weeks 3 and 4) revolved around coming to terms with the change in the look and feel of the penis, discomfort in doing ordinary things, and the initiation of sexual feelings. Over this time there was beginning of a return to normal especially as the pain and discomfort reduced. The soreness was still there, however, and so any arousal was still painful. Respondents developed their own ways of trying to deal with the pain and prevent themselves getting aroused.

*“At first*, *I didn’t like the way my penis looked*, *all bloated and red*. *Only after a few weeks*, *it started looking okay*.*” (Male*, *Muslim*, *34 years)*

*He used to keep a cold tin of Coke in the fridge and in the morning he would be sore and then he would keep the tin down there to get it down (laughs)*.*” (Female*, *Muslim*, *30 years)*

Participants generally reported no sexual activity in the first three weeks with some painful erections towards week three. Some reported masturbatory activities and oral sex from week four and there were a few who reported incidences of penetrative sex at weeks five and six. Although the penis remained very sore, they were able to have some form of sexual contact. When prompted, none of the participants mentioned condom use during this period.

*“No she did it with her hand and she was careful with the wound…” (Male*, *Muslim*, *27 years)*

*“I know I shouldn’t have done it*, *but we came from a party and we both had way too much to drink*, *and it just happened*, *you know…it was bloody sore afterwards and I tore the scabs a bit*, *but it was okay afterwards*.*” (Male*, *Christian*, *35 years)*

Participants who developed complications such as infection, swelling or wound tearing were generally happy with the post-operative staff and after the procedures in place. These complications were all reported in the first three weeks following surgery. Standard procedures are in place in the clinics for the VMMC men to return after three and seven days so the clinic staff could check the wound. All participants reported that they were told to go to the clinic in case of complications and all who reported a medical complication reported that they went back to the clinic.

*“I had an infection around the wound in the first week*, *but the clinic sorted me out nicely*.*” (Male*, *Muslim*,*22 years)*

### Non-penetrative sexual behaviour

In order to explore potential post-operative counselling topics, we asked participants about how they dealt with the recommended abstinence period when sexual desire returned and penile pain faded. During weeks four to six, participants reported engaging in some non-penetrative sexual behaviour such as kissing, fondling, finger penetration and oral sex. There was an interesting distinction between how men and women viewed these behaviours. Men generally viewed them as poor substitutes to ‘real sex’ and they expressed relief when they were allowed to engage in penetrative sex after the wound-healing period. Women, on the other hand, expressed gratification with non-penetrative sex where the emphasis was no longer on penile penetration. The “non-penetration-rule” almost forced the male to consider their partner as a sexual being with multiple erogenous areas other than only her vagina. It cultivated a greater awareness between the partners which was positively identified by the female partners.

*"Just because you're not having penetrative sex does not mean you are not having sex*.*" (Female*, *Christian*, *28 years)*

*“Things just got hot and I had to use my fingers and my mouth to satisfy her*. *It was difficult…” (Male*, *Muslim*, *21 years)*

*We were much more aware of ourselves*, *you know*? *Rather than concentrating on getting it on*, *we got to know one another more…learnt other parts of our bodies were also there…” Christian female*, *32 years)*

### Changes in penile sensitivity

Many participants reported a change in the physical act of sex. One spoke about the perception that exists in the community that circumcision is linked to loss of sexual capacity. Both men and women reported the men having lower sensitivity and taking longer to achieve orgasm. This was felt to be both positive and negative. Some felt that the decrease in penile sensitivity helped men with premature ejaculation difficulties, but it also aggravated erectile dysfunction problems in others. Women reported having to work harder in maintain their partners’ erection. This heightened the insecurities around their own sexual identity

*“…told me that he heard from a friend … that circumcision actually takes all the feeling away down there and that this person actually couldn’t get it up anymore*. *Naturally I was worried about that…” (Male*, *Muslim*, *19 years)*

*“It was okay*, *it wasn’t as nice as before*, *but I guess you get used to it…” (Male*, *Muslim*, *18 years)*

*“He took a lot longer to be excited*, *you know*, *get ready” (Female*, *Muslim*, *42 years)*

*“…much better now*, *I can control it better*.*” (Male*, *Christian*, *21 years)*

*“He looks much cleaner and it lasts much longer…” (Female*, *Christian*, *34 years)*

The issue of wearing condoms, however, did not change. When prompted, very few participants reported regular condom use, even those who professed to risky sexual behaviours such as casual and multiple partners.

*“(laughs) Condoms*? *Why*?*” (Male*, *Muslim*, *21 years)*

*I know it [condoms] protects and all that*, *but we are not sleeping around*, *so we don’t need it…’ (Female*, *Muslim*, *29 years)*

### Sex in the relationship

Difficulty in abstaining in the post-operative period has been identified as reason for early resumption of sex [15]. Therefore, in this study we also explored how important sex was for couples. We found that the sexual act plays a very important role in the participants’ relationships. It speaks to gender identity, desire, fear of partners being unfaithful and the projection of sexual needs on to partners. To the men, virility is linked to manhood so the need to keep women from being unfaithful feels a bit like wanting to maintain their property. Women also emphasised the role of sex in their relationships, albeit linked to partnership security and keeping the partner sexually satisfied. Both genders reported a need to use sex to maintain the relationship and keep their partner happy and interested.

*“If you don’t satisfy her*, *you can be sure that one day she is going to look somewhere else*.*” (Male*, *Christian*, *39 years)*

*“Men (laughs*.*) We have to keep them happy…” (Female*, *Muslim*, *47 years)*

*“You can never be in α relationship without having sex*.*” (Male*, *Christian*, *52 years)*

*“Yes*, *sex and love work together*.*” (Female*, *Muslim*, *25 years)*

A majority of our participants’ comments indicated that men and women believe that they are very different sexual beings. A message that recurred throughout our focus group discussions was that men are more sexually driven than women, and that women have an innately lower libido than men. The purpose of sex was also perceived to be different for men and women. Men spoke more about the raw physical drives of sex, with some mentioning that self-gratification is sought almost at the expense of the partner. Women referred to the sexual act as a component of a relationship.

*“It’s that male/female thing*. *For men I think it is just … well*, *it’s physical*. *It’s like they build up that sperm and they need a release” (Female*, *Muslim*, *22 years)*

*“I guess for men*, *if you could satisfy yourself*, *you don’t care about the girl*.*” (Male*, *Christian*, *31 years)*

*“I always felt like my husband was more of a sexual person than I was*, *but I always thought that was because men were more hot-blooded and wanted instant pleasure more than women*.*” (Female*, *Muslim*, *52 years)*

Finally, the comment cited in the title–“What do you mean I’ve got to wait six weeks?!”–was made by one of the participants during an informal chat at the end of a session (outside of the focus group setting), referring to his reaction when the health worker informed him about the no-sex restrictions during the wound-healing period.

## Discussion

In this study we sought to understand the experiences of the post-operative period after receiving VMMC of men and their female partners, from a primarily Coloured population, Participants in this study reported their primary motivation for seeking VMMC as being religious injunctions or for health benefits. Those men who were to convert to Islam as adults had decided to undergo the procedure within the first year after conversion. Islam as well as Judaism, stresses the hygienic benefits of circumcision as the main reason for its emphasis, although this was not voiced by the participants.

The issue of free choice is a dominant theme in the groups. It appears that very few of the Muslim men who came for VMMC did it by personal choice. They came because of religious requirements, partners need or health service insistence. This lack of choice might influence the male’s sexual behaviour over the six weeks as well as his willingness to adhere to rules including the non-penetration rule.

Among the non-Muslim participants a dominant reason for undertaking the procedure was hygiene. Both men and women named the presence and smell of dirt and semen trapped under the foreskin as motivation. Cleanliness is often seen as being the opposite of diseased [23], so it might not be seen as a specific attempt to reduce STIs, but there may have been a subliminal link to becoming disease-free.

VMMC is seen as an important tool in the HIV prevention toolkit. In a study conducted in 2012 in urban Swaziland the authors found that male circumcision, in general, is likely to foster protective behaviour change such as more responsible attitudes towards safe sex, reducing sexual temptation and partners and easier condom use [24].Although none of the participants in the present study cited HIV prevention as a reason for undergoing the procedure, our analysis found a number of men were encouraged to seek circumcision due to their high exposure to STIs. Although many participants knew that circumcision would provide some protection against HIV, there was a sense in the groups that this was not enough reason to get circumcised. With a rising HIV prevalence in the Coloured population, and the study participants’ stated aversion to condom use [25], there remains a heightened risk to HIV and other STIs. Racial communities in South Africa are still separated from each other in many ways, particularly in sexual relationships. The phenomenon of ascribing the HIV epidemic as a disease of ‘other race groups’ serves to perpetuate undue risky behaviour and stigma towards other groups. Thus, it may be that participants were unwilling to cite HIV prevention as a reason for male circumcision, even if this was a motivator. The alternative, that they really do not see themselves at risk of HIV, is more worrying, given the generalized nature of the epidemic in the Coloured population.

We also sought to address the phenomenon that has been reported in other settings [24] that men, especially men in committed relationships, engage in penetrative sex before the recommended wound-healing period of six weeks has been completed. We indeed found that after 4–5 weeks of no ‘real’ sex, tensions arose due to several factors. As described above, couples live in fairly densely populated areas in small and cramped houses. Living in very close quarters with each other with very little interpersonal space, makes it difficult to avoid sexual arousal and the need for sexual gratification. The real drive for sexual contact is obviously very high. Additionally, men felt pressure from the socially-induced fear of failing to satisfy their partners and therefore not maintaining the harmony of the relationship. These factors lend a powerful motivation to forgo the ‘no penetration’-rule. Thus, some men did report penetrative sex during this period. All reported instances of penetrative sex were either fuelled by alcohol or an escalation of non-penetrative sessions, where the normal sexual patterns between the two partners overrode the ‘temporary’ restrictions placed on them by the procedure.

We also wanted to gain a better understanding of the interpersonal and cultural constructions of gender identity and how they relate to sex and sexuality. Such knowledge could potentially aid in developing more appropriate VMMC counselling interventions. Thus, we looked at the perceptions of men and their partners on having to withdraw from penetrative sex in the post-operative period on the men and their partners and on their relationships and the forms of non-penetrative sex practised and other ways of stabilising the relationship. Fundamental to this was how men and women perceived the role of sex in relationships.

Although the departure points are different for both genders, the importance of sex in the relationship was highlighted by both. This difference speaks directly to issues of gender identity. Whereas the males saw sex as a physical act that confirmed masculinity, many males saw the intimacy as a mechanism to maintain the relationship. The role as the provider, whether it was sustenance, physical items or intimacy, was paramount toe men’s understanding of what it means to be a man. Women, on the other hand, saw sex more as an affirmation of a relationship and their role to provide a home for the ‘baser’ needs of the man.

The discussions indicated that both men and women in this population saw sex as essential to a good relationship and that men are perceived to be more sexual than women. Moreover, men commonly experienced conflict around sex and used emotions to manage their own and their partners’ feelings about sex. A general theme around sex that emerged in the analysis was that men and women view sexual activity as a gauge of marital success.

The importance that couples place on sex in relationships means that it is essential to understand how couples negotiate the six-week abstinence period, particularly the last two weeks. The participants of this study said they engaged in non-penetrative sexual activity, such as rubbing, mutual masturbation, kissing or cuddling that they employed during this period. Some even included penetrative aspects such as with a finger or the mouth in oral sex. The participants reported using some of these methods as alternatives to penile penetrative sex. There was a general feeling among male participants, however, that it was a poor substitute for ‘real sex’.

We also asked about issues related to penile function after VMMC as it was linked to erectile dysfunction. The sensitivity of the cut penis had both negative and positive comments. Negative, because it was not as responsive or pleasurable as before and that influenced erectile duration and quality especially in the older men. It was also positive because circumcised men took longer to reach ejaculation, which was viewed as an advantage by both genders, rather than a complication. These issues would benefit from further explanatory research.

### Implications for public health practice

The results of this study have relevance for guiding the design of VMMC services in the Cape Town area. Findings could be of relevance for the recruitment of Coloured populations to VMMC, which is an important tool in the health department’s HIV prevention toolbox. The Coloured population does not seem to be motivated to undergo VMMC for HIV prevention reasons. On the other hand “STI prevention” and “hygiene” seem to be acceptable motivators. Thus, recruitment campaigns for VMMC in this province may be more successful if it is not promoted as “HIV prevention.”

Our results also point to potential topics to be included in pre- or post-operative counselling for VMMC patients. First, given the centrality of sexually conjugal relations in the population accessing the services, and the frustrations expressed with the post-operative abstinence period, it seems advisable to discuss this topic with the patient. One possibility is helping the patient to develop a plan for how he (and his partner) will negotiate the recommended abstinence period. Alternatives to penetrative sex should be discussed. Avoidance of alcohol and drugs, which may induce sexual arousal, during this period should also be discussed.

Finally, the number of participants who did not think that condom use was important is worrisome. The necessity of condom use to avoid transmission of STIs between partners should be stressed in counselling. This is, of course, particularly important for those patients who are found to be HIV positive or who are referred because of frequent STIs.

The effectiveness of counselling in behavioural change is varied. VMMC services are relatively new and often carried out in settings with limited human resources. Long counselling sessions are often not realistic. In some areas of South Africa, such services are being carried out by independent actors holding “vaccination camps.” It is essential from a public health, and gender, perspective that effective counselling strategies for the VMMC post-operative period, and the longer term, are developed and tested.

## Limitations of study

The study has several potential limitations. Firstly, we recorded the responses of only a small number of participants in a somewhat artificial environment. We cannot rule out the possibility that participants may have felt pressured to respond in certain socially acceptable ways. We tried to minimise this by stressing confidentiality, and that their fellow participants went through the same experiences. Secondly, the female groups were facilitated by a male facilitator. Although the facilitator is very experienced and went out of his way to create a safe environment, this may have hampered spontaneous discussion on fairly sensitive topics. For example, female respondents may not have felt comfortable describing sexual frustration during the recommended abstinence period to a male interviewer, and thus resorted to explanations that are more firmly rooted in socially acceptable norms. Thirdly, for logistical reasons, the focus groups were held in the month of Ramadan, which also potentially hampered candid discussions on sex and relationships for the Muslim participants. Fourthly, data on HIV status and or having a long-term versus casual partner, which may affect perceptions of MMC or sexual behaviour in the post-operative period, was not collected. Such information could have added additional depth to the analyses. Finally, we do not know to what degree the sample we recruited was representative, but it is likely that the high representation of Muslims is a reflection of the population seeking VMMC services in the area.
